# Artificial Intelligence in Clinical Nutrition: Current Uses, Challenges, and Opportunities

**DOI:** 10.3390/nu18162638

**Published:** 2026-08-12

**Authors:** Kasuen Mauldin, Anthony D. Pham, Sneha Dodaballapur, Berkeley N. Limketkai

**Affiliations:** 1 Clinical Nutrition, Stanford Health Care, 300 Pasteur Drive, H1207, Stanford, Palo Alto, CA 94305, USA; 2 Nutrition, Food Science, and Packaging, San José State University, One Washington Square, San José, CA 95192, USA; anthony.pham1756@gmail.com; 3 Department of Medicine, Los Angeles Medical Center, Olive View-University of California, 14445 Olive View Drive, Sylmar, Los Angeles, CA 91342, USA; sdodaballapur@dhs.lacounty.gov; 4 Division of Digestive Diseases, University of California Los Angeles School of Medicine, Los Angeles, CA 90095, USA; berkeley.limketkai@gmail.com; 5 Division of Clinical Nutrition, University of California Los Angeles School of Medicine, Los Angeles, CA 90095, USA

**Keywords:** artificial intelligence, Nutrition Care Process, clinical nutrition, personalized nutrition

## Abstract

Artificial intelligence (AI) is increasingly being applied in healthcare, with growing relevance to clinical nutrition. This narrative review examines current and emerging uses of AI in nutrition care within the Nutrition Care Process framework, with attention to assessment, monitoring and evaluation, diagnosis, intervention, and clinical support tools. Current applications include AI-assisted dietary assessment using image recognition, wearable sensors, analysis of continuous glucose and other physiologic data for early risk detection, and support for malnutrition screening and diagnosis. AI is also being explored for identifying micronutrient deficiencies and complications of nutrient excess, as well as for screening and early intervention in eating disorders. In nutrition intervention, AI has potential to support personalized dietary planning, nutrition support in intensive care settings, behavioral interventions, and precision nutrition approaches such as digital twins. Additional applications include clinical decision support and documentation assistance. However, despite its usefulness, concerns about AI systems exist. Its performance depends on the quality of the data used to train it; it can introduce bias, and it can produce inaccurate or misleading outputs. In addition, overreliance on AI may also reduce clinician attentiveness and contribute to cognitive errors. For these reasons, AI should be regarded as a support tool rather than a replacement for human clinical care. Overall, AI offers substantial opportunities to improve the personalization, efficiency, and scalability of clinical nutrition practice, but its safe and effective implementation will require continued validation, careful oversight, and integration with clinical expertise.

## 1. Introduction

Artificial intelligence (AI) has advanced rapidly in recent years, driven by accelerating increases in computing power and the availability of large datasets for model training. As data volume and quality improve, AI systems become more accurate and better able to generate meaningful, user-centered outputs. AI has quickly transformed some industries such as finance and retail, yet its integration into clinical care, particularly in clinical decision-making, has progressed more slowly. Although healthcare is increasingly digitized and many clinical outcomes are quantifiable (i.e., large datasets for AI model training), healthcare faces unique challenges, including privacy concerns, algorithmic bias, regulatory complexity, liability risk, and the need for ongoing human oversight [1,2,3].

Examples of current AI uses in healthcare in general are presented in Table 1. All the examples highlighted use AI to turn large amounts of data into actionable support for healthcare. These examples are data-driven, designed to assist decision-making rather than replace clinicians [2,4], and aimed at making clinical care more personalized, efficient, and scalable.

Several recent reviews examined the role of AI in nutrition. Some have provided broad overviews of AI applications across clinical nutrition and dietetics [3] or across the wider field of nutrition science, including food science and the public health realm [22], while others have focused on machine learning and deep learning models to improve nutrition-related screening and outcomes [23] or on the emerging role of generative AI in clinical decision-making for common, chronic conditions [24]. However, existing reviews have generally not organized AI applications according to the structured, iterative framework used by clinicians to deliver nutrition care, nor have they addressed AI applications in gastroenterology and nutrition-related disease management, clinical support and documentation tools, or the medicolegal considerations.

This narrative review examines the current and emerging roles of AI in clinical nutrition care, within the Nutrition Care Process (NCP) framework, highlighting case examples of AI in medical nutrition therapy for various nutrition-related conditions. This review is designed to move beyond technical descriptions of AI tools to critically evaluate their clinical utility, limitations, and future potential in managing common nutrition-related diseases.

## 2. Methods

The references used for this narrative review were selected from targeted searches of PubMed and Google Scholar. Articles were selected for their relevance to clinical nutrition practice, the Nutrition Care Process, and nutrition-related medical conditions. More recent studies, published within the past five years, were prioritized to align with the timeline of AI growth. However, earlier AI research studies, especially those that provided background for understanding current applications, were also included. We also reviewed references from relevant articles to identify additional publications. Because this is a narrative review, the search was intended to identify representative and clinically relevant literature rather than provide a comprehensive or systematic review.

During the preparation of this manuscript, the authors used generative AI (ChatGPT-5.5, OpenAI, San Francisco, CA, USA) to generate and edit the graphical abstract and the figure. Generative AI tools (Claude-Sonnet 5, Anthropic, San Francisco, CA, USA and ChatGPT-5.5, OpenAI, San Francisco, CA) were also used to assist in organizing and synthesizing information based on the author-written content in the manuscript into a draft summary table. The authors have reviewed, edited, and added to the outputs and take full responsibility for the content of this publication.

## 3. The Use of AI Across the Nutrition Care Process Model

The Nutrition Care Process model is the Academy of Nutrition and Dietetics’ framework for delivering nutrition care [25]. It includes four core steps: nutrition assessment, diagnosis, intervention, and monitoring and evaluation. The model also incorporates nutrition screening and outcomes management, which are part of the overall framework but occur outside the core NCP steps. AI tools are increasingly being integrated across each component of this model [26].

The NCP is inherently connected through the continuous use of the same underlying data across all steps, which is particularly relevant when considering AI applications, as these tools are most effective in analyzing and integrating large, complex datasets. Data collected during nutrition assessment, such as dietary intake, anthropometrics, laboratory values, and other clinical indicators, form the basis for identifying a nutrition diagnosis. While this information often overlaps with medical data used to establish a medical diagnosis, the nutrition diagnosis focuses specifically on nutrition-related problems, which may differ in scope or emphasis from the medical condition. The nutrition diagnosis is made by a registered dietitian (RD) and focuses on how nutrition is affected by or contributing to the medical condition. For example, a medical diagnosis is “chronic kidney disease,” while the nutrition diagnosis might be “inadequate protein intake” or “excess sodium intake” [27].

The data used in nutrition assessment is carried forward into monitoring and evaluation, where changes in those indicators are used to determine whether nutrition interventions are effective. In this way, the NCP functions as a feedback loop, with assessment and reassessment data informing nutrition diagnosis, guiding intervention, and providing measurable outcomes for nutrition monitoring and evaluation. AI is particularly well suited to this model because it can efficiently integrate and analyze large, complex datasets, supporting clinicians in identifying nutrition problems and tracking progress over time. Current applications of AI in nutrition care reflect this strength, focusing on synthesizing assessment data to inform diagnosis (both nutrition and medical diagnoses) and using the same data streams to monitor intervention outcomes.

## 4. AI Applications for Nutrition Assessment, Monitoring and Evaluation

AI-powered dietary assessment tools have the potential to use image recognition, wearable sensors (detecting jaw motion and chewing patterns), and natural language processing to estimate food and nutrient intake more accurately than traditional self-report methods [28]. A recent scoping review of AI tools in assessing food and nutrient intakes found that food detection accuracies range from 74% to 99.85%, with nutrient estimation errors between 10 and 15% [28]. In addition, a systematic review comparing AI-based digital image dietary assessment methods with humans found that AI can estimate food volume and calories about as well as, and sometimes better than, humans [29]. However, differences in the food image datasets and how results were reported made it hard to compare studies. The field of image-based dietary assessment could be improved by using a few large, standardized datasets to train and test AI models [29].

Beyond model accuracy, several practical challenges continue to limit clinical implementation. Image-based systems may have difficulty identifying mixed dishes, recipes with hidden ingredients (e.g., oils, sauces, seasonings), or foods that appear visually similar but differ substantially in nutrient composition. Accurate portion size estimation remains challenging because it depends on image angle, lighting, occlusion, and the presence of reference objects. Performance may also decline when models are applied to culturally diverse foods or regional cuisines that are underrepresented in training datasets. Furthermore, nutrient estimates depend on the completeness and quality of the underlying food composition databases, which may vary across countries and may not fully capture branded or restaurant foods. These factors can introduce clinically meaningful errors in nutrient estimation, particularly when precise dietary assessment is required for conditions such as diabetes mellitus, chronic kidney disease, or nutrition support. Continued development of standardized image datasets, more representative food databases, and prospective validation in diverse clinical populations will be important before these technologies can be widely integrated into routine nutrition practice.

AI is being applied to wearable devices and continuous glucose monitors (CGMs) to shift healthcare from reactive to predictive. AI-enabled wearables have been deployed for the detection of arrhythmias, sleep apnea, depression, and chronic disease progression [14]. For example, using AI to analyze CGM data in patients with prediabetes can identify subtle blood glucose fluctuation patterns, predict postprandial glucose responses, and potentially generate personalized dietary and lifestyle recommendations before blood glucose values ever reach diabetes-diagnostic levels [15]. However, challenges remain: the evidence base remains largely conceptual, most accuracy values come from small or single-center studies, and standardized databases and reporting methods do not yet exist. In addition, cost remains a barrier, particularly in low-resource settings where the burden of diet-related chronic disease is highest [14].

Another area where AI shows promise is monitoring laboratory levels, particularly potassium levels. High potassium can be life-threatening if not managed quickly. AI can detect signs of high potassium using heart data, without needing a blood test. This could allow for faster response, including adjusting a patient’s diet before the condition becomes dangerous [11,30]. These AI systems have the capability to detect patients with kidney disease, including those receiving dialysis.

## 5. AI Applications for Nutrition-Related Conditions

To diagnose a nutrition-related disease, AI can leverage several approaches: computer vision to assess observable physical manifestations of disease; analysis of clinical and laboratory abnormalities; and disease prediction based on clinical risk factors. In the context of nutrition, these functions have potential application for the diagnosis of undernutrition (e.g., protein-calorie malnutrition, nutrient deficiencies) and complications of nutrient excess (e.g., metabolic syndrome, steatotic liver disease).

### 5.1. Malnutrition

Scoring systems for the identification of malnutrition are generally divided into two roles: screening and diagnosis. Screening tools, such as Nutritional Risk Screening 2002 (NRS-2002) and Malnutrition Screening Tool (MST), rely on patient-reportable data (e.g., anthropometrics, reduction in oral intake, weight loss, and acuity of illness) to estimate risk of malnutrition [31,32]. By contrast, tools for diagnosis of malnutrition (discussed in a subsequent section), such as the Subjective Global Assessment (SGA) and the Global Leadership Initiative on Malnutrition (GLIM) criteria for malnutrition, additionally require physical examination findings [33,34].

#### 5.1.1. Malnutrition Screening

The use of patient-reportable criteria in malnutrition risk screening tools facilitates remote and continual data collection. The simplicity of these tools obviates the need for AI to analyze reported data. On the other hand, AI may still provide benefit in several scenarios, such as (i) the analysis of patient data when missing data preclude calculation of a full score (e.g., patient forgets to provide data on relative food intake); (ii) consideration of additional clinical variables (beyond the simplified scoring criteria) to improve the sensitivity and specificity of malnutrition risk assessment; and (iii) assessment in specific subpopulations (e.g., age groups, different diseases), as scoring systems may not perform uniformly well across such populations. Scoring systems often rely on complete responses to each criterion to calculate a final score and provide a risk estimate; it is therefore highly sensitive to missing data. However, AI systems can compensate for missing data by leveraging other clinical variables from the electronic health record (EHR) to still make a probabilistic estimation of malnutrition risk. Moreover, the availability of these collateral data can augment the performance of risk prediction. In a study of 106,449 hospitalized patients in the United States, an AI-based malnutrition screening pipeline was compared with the nurse-administered MST [35]. The AI system used a large language model (LLM) for feature extraction from the EHR, followed by use of machine learning (ML) gradient-boosted decision trees to classify malnutrition risk. Using discharge-coded malnutrition as the outcome, the sensitivity, specificity, and positive predictive value (PPV) were 0.49, 0.97, and 0.55, respectively, for the AI system, compared with 0.24, 0.95, and 0.27, respectively, for the MST. Using dietitian-recorded malnutrition as the outcome, the metrics were 0.52, 0.96, and 0.37, respectively, for the AI system and 0.35, 0.95, and 0.25, respectively, for the MST. These differences demonstrate how incorporation of additional relevant clinical variables can boost prediction performance. Consideration of different diseases and subpopulations, such as in elderly patients hospitalized with congestive heart failure or children undergoing surgical repair of congenital heart disease, further highlights the advantages of AI over traditional scoring systems [36,37].

Because AI can process large volumes of data autonomously, efficiently, and at scale, AI has already enhanced the collection of data for nutrition screening and assessment in practice. For example, Malnutrition Universal Screening Tool (MUST)-Plus, an ML-based malnutrition screening tool, has been implemented in some hospital workflows, analyzing EHR data to identify malnourished patients with high accuracy [38,39,40]. These tools exceeded 90% usability among RDs and reduced the lag time between admission and malnutrition diagnosis [39]. Deep learning (DL) models predict malnutrition from longitudinal patient records, outperforming traditional screening instruments while requiring less data collection [38].

#### 5.1.2. Malnutrition Diagnosis

Unlike with risk screening, formal diagnosis of malnutrition requires data on body anthropometrics. The GLIM criteria mandate that muscle mass be assessed by validated measures, such as physical examination, mid-upper arm or calf circumference, dual-energy absorptiometry, bioelectrical impedance analysis, computed tomography (CT), or magnetic resonance imaging. This additional step introduces an opportunity for AI to improve clinical workflows. For example, to develop a semi-autonomous system for diagnosis of malnutrition, computer vision could be used to provide insight into body composition. As cross-sectional CT images of the lumbar or thigh muscles are validated indicators of skeletal muscle mass, AI could perform auto-segmentation of relevant CT image slices and measurement of muscle mass [41,42,43]. In a study of 3096 cases with lumbar CT imaging, the DL model had a sensitivity, specificity, and PPV of 82.3%, 98.1%, and 89.5%, respectively [44]. DL models can similarly be applied to ultrasonographic images, which are more convenient than CT and could technically be performed at the point of care [45,46]. Compared with CT of the lumbar musculature, ultrasonography of the rectus femoris has modest correlation [47].

AI systems have been developed for image- or video-based examination of skin cancer, inflammatory skin diseases, gait impairments, and others [48,49,50,51]. Such functions can similarly be extended to analyze images to assess muscle mass or sarcopenia. While automated analyses of CT or ultrasonographic data can aid assessment of muscle mass, the patient must still undergo on-site testing. These modalities also present additional cost, increased demand on limited imaging resources, radiation exposure (for CT), and need for specialized equipment or software. Moreover, validation studies are still needed prior to deployment of these technologies in the clinic. One could easily argue that these modalities do not provide distinct advantages beyond the quicker and more convenient physical examination performed by the clinician.

### 5.2. Diagnosis of Micronutrient Deficiencies

Micronutrient deficiencies are generally diagnosed via laboratory testing. However, initial suspicion of a micronutrient deficiency based on clinical context or detection of related complications should alert the clinician to pursue laboratory investigation. The opportunity for AI systems to provide value in this scenario is with risk prediction (assessment of probable micronutrient deficiency and alerting clinicians to consider laboratory testing) rather than test interpretation (after the test has been performed). Risk prediction can occur via analysis of aggregate symptoms and/or clinical data from the EHR. For instance, if a patient presents with hair loss, dermatitis, loss of taste or smell, and poor wound healing, a zinc deficiency would be suspected. If a patient were to present with bleeding gums, easy bruising, poor wound healing, and corkscrew hair, a vitamin C deficiency would be suspected. AI currently has the technical capability of making such assessments via LLMs, where the user can enter a constellation of symptoms to generate a differential diagnosis. There is an even greater dearth in using computer vision to diagnose micronutrient deficiency. One study reported the development of AI-assisted evaluation of wrist radiographs to detect rickets with good sensitivity (80%) and specificity (95%) [52].

In the meantime, the use of AI related to micronutrient deficiency has largely relied on analysis of indirect data for risk prediction. In a cohort of 51,630 adults, ML and DL models examined routine laboratory tests (i.e., complete blood counts, comprehensive metabolic panel) to predict vitamin B12 deficiency [53]. The best-performing model had an area under the receiver operating characteristic curve (AUROC) of 0.88, sensitivity of 0.92, specificity of 0.67, and PPV of 0.73. Such models would be useful for guiding selective laboratory testing. In a separate study, DL was used to predict iodine deficiency in the first trimester of pregnancy with modest sensitivity (0.56) and good specificity (0.85) [54]. The DL model had 12 features: age, body mass index (BMI), number of gestations or gestation week, iodine supplementation, iodized salt intake, folic acid intake, consumption of several foods (i.e., milk, fish, crustaceans, mollusk, seaweed), and residence. Unlike with the models to predict vitamin B12 deficiency, the DL model to predict iodine deficiency required specific data that are not routinely collected in clinical care. As such, beyond consideration of model performance, required changes in the clinical workflow need to be considered and whether such changes are worthwhile; routine laboratory testing may sometimes be more efficient. There are nonetheless potential circumstances where semi-automated prediction of micronutrient deficiencies would still be beneficial, such as in resource-constrained settings or broad population-based health surveillance.

In a study of 5106 adults between 50 and 84 years old, ML models were constructed to predict vitamin D deficiency based on two sets of features [55]. One set of “simple” features included demographic data, select diagnoses (i.e., hypertension, diabetes, cardiovascular disease), treatment history (i.e., urgent treatment for asthma or chronic bronchitis/emphysema, antihypertensive medication), smoking status, BMI, and systolic blood pressure. The “augmented” set of features included data on marital status, general health, vitamin D supplementation, sun exposure, physical activity, alcohol intake, serum albumin, and creatinine. The ML models generally performed better with the “augmented” model and an outcome of 25-hydroxyvitamin D < 25 nmol/L. Another study used data from 62,919 individuals in the 2001–2018 cycles of the National Health and Nutritional Examination Survey to develop ML models to predict vitamin D deficiency [56], and some models performed very well (e.g., extreme gradient boosting had an AUROC 1, sensitivity 0.998, specificity 0.999, PPV 0.998). Features included age, race, sex, household size, household income-to-poverty income ratio, BMI, household smokers, past 30-day milk product consumption, and diabetes. While these studies provide proofs of concept of predictive modeling based on indirectly related data, the need for less readily accessible data limits their practical benefit. In addition, the near-perfect predictive performance may mask technical or practical issues, such as model overfitting, lack of external validation, and poor generalizability to subpopulations or other populations. Nonetheless, such technologies can still be useful for population-based characterization of risk factors and estimation of prevalence of vitamin deficiencies [56,57,58].

### 5.3. Diagnosis of Nutrient Excess

AI-assisted diagnosis of overnutrition-associated diseases can adopt similar concepts as those for undernutrition-associated diseases. Although the diagnosis of obesity is often simply inferred with straightforward anthropometric measures, detection of underlying sequelae of visceral adiposity is more elusive. In this scenario, AI models could theoretically be used to identify at-risk populations or support diagnoses without the need for more involved testing. Hepatic steatosis is often diagnosed via imaging modalities, such as ultrasound, CT, or magnetic resonance imaging. ML has been tested to predict hepatic steatosis using demographic and laboratory values [59]. Although the best-performing model had good specificity (94.6%), sensitivity was more modest (45.2%). The highest sensitivity achieved by any model was 68.0%. These observations highlight some limitations of diagnostics using indirect data. While AI can improve workflows for convenience, data quality and system performance are critical considerations prior to deployment in clinical practice.

Beyond diagnosis, AI could also serve a role in future risk prediction. ML models to predict obesity among adolescents have been developed using sociodemographic characteristics, academic performance, and physical activity [60]. The models had modest performance with AUROC 0.68 to 0.75, and the most prominent predictors of obesity included gender, sedentary time, school location (e.g., urban, suburban, rural), and birthweight. While such AI tools could be helpful for population-based disease risk assessment, modest performance may limit their ability for individual-level risk prediction. Nonetheless, the broader question remains on utility: would it be more efficient and effective to focus public health investment on promoting increased physical activity in schools?

### 5.4. Medical Nutrition Therapy for Eating Disorders

Nutrition screening is the earliest point in the NCP model where AI may add value by helping identify patients at risk before more advanced nutrition problems develop. AI-assisted apps are being designed to screen for eating disorders. These apps detect early signs of problematic eating behaviors by assessing patterns over time using self-reported data [61]. By tracking daily food intake, exercise habits, and emotional states, these apps can flag unusual patterns that may suggest the onset of an eating disorder, with some platforms incorporating ML algorithms to analyze user data and provide personalized feedback. For example, Ioannidis et al. designed an AI model to predict potential diagnoses of anorexia nervosa. The early detection system was trained to analyze 16 physiological parameters including BMI, heart rate, oxygen saturation, and blood panels in anorexia nervosa patients within the United Kingdom [62]. Simultaneous to these assessments, the AI model monitored other biomarkers like potassium, sodium, and phosphorus and used these variables within its anorexia nervosa risk classification process [62]. This model produced an AUROC of 0.92 compared to an AUROC of 0.89 in control groups [62]. Inclusion of AI models as part of NCP screening and diagnosis allows for improved detection and earlier intervention of eating disorder-associated behaviors.

One of the more technical applications of AI involves combining brain imaging with ML to identify neurobiological markers of eating disorders. Studies utilizing magnetic resonance imaging have shown success in distinguishing anorexia nervosa patients from healthy controls, identifying brain regions involved in anorexia nervosa pathophysiology, and even predicting long-term outcomes. AI has the capacity to scan patterns in the brain and analyze for signature characteristics of anorexia nervosa [63]. Researchers found support vector machines to exceed accuracies of 80% in regard to predicting anorexia nervosa diagnosis. For example, an AI system was trained to identify specific brain regions associated with anorexia nervosa and estimate the likelihood that a patient would belong to the anorexia nervosa group or normal control group. AI models observed that the right putamen, third ventricle, and right ventral diencephalon had comparably more volumetric reductions compared to the left cerebellum [64]. The model correctly predicted with 83.3% accuracy (n = 30) that an individual is diagnosed with anorexia nervosa [64]. Other machine learning models were used to analyze hair data, patient statements, social media posts, and medical reports to detect early signs of anorexia nervosa [65]. For clinicians, this early screening can prompt immediate referrals and timely intervention in order to prevent further malnutrition [63].

### 5.5. AI Applications in Gastroenterology and Nutrition Research

AI has found multiple roles in gastroenterology, such as AI-assisted polyp detection, assessment of colonoscopy surveillance intervals, recommendations for surgical management of gastroesophageal reflux disease, and prediction of inflammatory bowel disease (IBD)-related healthcare utilization [66,67,68,69]. Although there are overall fewer AI applications available at the intersection of gastroenterology and nutrition, progressive advances in this domain suggest significant future potential.

Through its ability to analyze large and complex datasets, AI has important benefits for research and discovery. In a study of 691 participants with IBD, ML methods were used to identify dietary patterns that were then associated with risk of symptom relapse [70]. The benefit of ML over traditional diet classification schemes is the ability to cluster diets without preconceived notions of “healthy” or “unhealthy” food types, while also categorizing habitual diet types that better mimic real-world behaviors.

In a separate study, we applied LLMs to automatically screen 13,000 articles for inclusion in a systematic review and meta-analysis of dietary interventions for IBD (unpublished data). Open-source models (gpt-oss-20b and gpt-oss-120b) had a 96% accuracy and performed similarly to ChatGPT 5.4’s 98% accuracy, highlighting the potential of AI for efficiently curating nutrition-related articles. These findings also indicate that more cost-effective open-source options may be adequate for more straightforward tasks. Nonetheless, these are unpublished data that must still pass the rigorous assessment of peer review and should not be considered with equal weight as other published evidence.

Analysis of diet-gut interactions is a frontier where AI shines. Its ability to examine complex interactions across vast data elements, while accounting for inter-individual differences, makes AI particularly well-suited for integrative analysis of clinical, dietary, and multi-omic (e.g., metagenomic, metatranscriptomic, metaproteomic, metabolomic) data. This feature enables researchers to uncover important microbial signatures, metabolic biomarkers, and mechanistic pathways in otherwise undecipherable datasets [71].

## 6. AI Applications in Nutrition Intervention

### 6.1. Dietary Interventions

Nutrition interventions such as the creation of personalized dietary plans for specific populations have benefited from AI assistance. For example, athletes have unique nutritional needs that are highly variable and closely tied to performance, unlike general populations where needs are more stable. Their dietary requirements change frequently based on training intensity, competition schedules, recovery status, and environmental conditions, requiring continuous adjustment rather than fixed recommendations. AI tools such as ML, computer vision, wearable biosensors, and LLMs are shifting athlete nutrition interventions away from generic guidelines toward individualized plans [12]. These plans are based on real-time data from CGMs, sweat analysis, heart rate variability, and multi-omics profiles [12].

AI is being used to support patients at home. Many patients have questions or concerns that are not urgent but still need attention. AI chatbots can help answer these questions and guide patients in their daily care [16]. Early results show that using chatbots may reduce infections and complications related to dialysis. This is important because infections can lead to further health problems and worsen nutrition by increasing protein loss [18,72]. This information is valuable for dietitians in order to create tailored nutrition interventions that can address inadequate protein intake. While these results are promising, they are inherently limited due to their geographic study designs. Therefore, more research is necessary in order to confirm how well these tools work across different patient groups. The main implications at this time would be AI’s potential for improving patient nutrition education and self-care.

AI may also support nutrition intervention in acute kidney injury (AKI) by enabling earlier recognition of patients who are likely to require prompt dietary modification. AI-based alert systems can identify patients at risk of AKI or clinical deterioration before the condition becomes severe [30,73,74,75]. From a nutrition care perspective, these early warnings may help clinicians initiate timely interventions to address protein needs and electrolyte abnormalities, particularly when potassium and other disturbances can become life-threatening if not managed quickly. In this way, AI does not replace clinical decision-making but may help make nutrition intervention more responsive to rapid changes in patient status.

In chronic kidney disease, LLMs such as ChatGPT are being explored as tools to support dietary intervention through generation of kidney-friendly recipes, menu translation, and analysis of food images for nutritional content [11]. These applications are directly relevant to dietary counseling because they may improve accessibility, personalization, and patient engagement. However, accuracy remains an area of concern, as evidenced by ChatGPT 4 underestimating calories by 36%, protein by 28%, potassium by 49%, and phosphorus by 54% compared to United States Department of Agriculture-validated software, and these errors in nutrient analysis can lead to serious clinical implications for patients with renal disease [11]. Such errors are likely multifactorial. Unlike dedicated nutrient analysis software, general-purpose LLMs are not directly linked to validated food composition databases and instead generate responses probabilistically based on patterns learned during training. Consequently, nutrient estimates may vary depending on prompt wording, the level of detail provided about foods and portion sizes, and the model’s ability to interpret mixed dishes, branded products, or complex recipes. In addition, incomplete or outdated food composition information and differences among regional food databases may further contribute to inaccuracies.

Given the potentially life-threatening consequences of nutrient miscalculations in patients with nutrition-related conditions such as chronic kidney disease, AI-generated dietary recommendations should be considered preliminary. Practical safeguards include having an RD or other qualified clinician review all AI-generated recommendations, verifying nutrient estimates against validated nutrient analysis software or established food composition databases, and confirming adherence to current evidence-based renal nutrition guidelines. Future improvements may include standardized prompting strategies, integration with validated food composition databases, and development of nutrition-specific AI models to enhance the usefulness of these systems.

AI outputs should also be interpreted within the context of the individual patient’s laboratory values, medications, dialysis status, and overall clinical condition, rather than being used as a stand-alone decision-making tool. As LLMs continue to evolve, prospective clinical validation across diverse patient populations and standardized benchmarking against reference dietary analysis methods will be necessary before these systems can be incorporated into routine renal nutrition practice. In summary, AI-generated dietary interventions currently require review and validation by an RD or other qualified nutrition professional before implementation.

### 6.2. Behavioral Modification Interventions

Therabot, a generative AI chatbot developed for management of mental health conditions such as depression and anxiety, was used in treating eating disorders [76,77,78]. Therabot development involved expert refinement by more than 70 specialists over 100,000 human hours, and it was specifically trained to deliver evidence-based interventions for these conditions. In a randomized controlled trial (RCT), participants who used Therabot for four weeks showed significantly greater reductions in eating disorder symptoms than waitlist controls, both immediately after the intervention and at 8-week follow-up [76,77]. In dietetics practice, early intervention for body dysmorphia, eating habits, and food perception can influence the severity of malnutrition and acuteness of disordered eating. In more severe stages of malnutrition, AI can analyze comprehensive metabolic panels alongside patient anthropometrics, which can be particularly useful for medical nutrition therapy in order to provide more personalized nutrition interventions. This can allow for timely initiation of early cognitive behavioral therapy in order to address behaviors and beliefs associated with eating disorders [64].

Despite these promising findings, AI-assisted interventions for eating disorders require caution because of the vulnerability of this patient population. Inaccurate or inappropriate chatbot responses, reinforcement of maladaptive eating behaviors or body image concerns, and incorrect psychological or nutritional advice could delay appropriate treatment or contribute to patient harm. Accordingly, AI should be viewed as an adjunct to, rather than a replacement for, multidisciplinary care. Clinician oversight remains essential to interpret AI-generated recommendations, monitor patient safety, and ensure that interventions remain consistent with evidence-based eating disorder management.

In a study examining clinician and community participant perspectives on incorporating AI into the clinical management of eating disorders, the majority of respondents were open to AI as a tool to support clinical care [79]. Although further validation and careful implementation are needed, these findings suggest growing acceptance of AI-assisted approaches and support continued research and development of AI applications in eating disorder management.

### 6.3. Nutrition Support Interventions

AI tools can allow dietitians to make more informed decisions regarding nutrition support interventions, especially for complex intensive care unit (ICU) cases. To date, AI has been more effectively applied to nutrition-related disease diagnosis and diagnosis of nutrition problems related to enteral nutrition (EN) support. For example, AI has been used to predict nutrition-related clinical events in intensive care settings, including enteral feeding intolerance, diarrhea, and refeeding hypophosphatemia [6,23,80]. AI has also been used to optimize feeding timing and assist in identifying candidates for total parenteral nutrition (TPN) when EN fails [81]. EN initiation currently depends heavily on medical team awareness and judgment, and there is an opportunity for ML algorithms to assist in early evaluation of EN [82]. For example, Wang et al. developed an AI model trained on over 53,000 patients to predict whether an ICU patient will require EN initiation [82]. The model analyzed routine clinical data within the first 24 h of admission in order to flag patients who require early feeding intervention [82]. They found that sepsis (followed by sequential organ failure assessment score, AKI, and body temperature) was the strongest predictor of EN initiation [82]. In addition to AI identifying patients needing early nutrition support, it can also proactively assist RDs with continuous nutritional status monitoring and flag patients that show signs of decline. This allows for immediate changes to nutrition support prescriptions that are better tailored for the patient’s current status.

More recently, a 2025 study introduced TPN2.0, an AI system designed to optimize TPN for newborns in neonatal ICUs [83]. Premature and critically ill newborns often rely on TPN because they cannot tolerate adequate EN. In this study, ML was used not just to predict risk, but to directly guide individualized nutrition therapy. The researchers trained TPN2.0 using 79,790 TPN prescriptions from 5913 neonates at Stanford Health Care and then conducted external validation on 63,273 prescriptions from 3417 infants at the University of California San Francisco Medical Center [83]. TPN2.0 used laboratory values, demographics, growth data, medications, procedures, and other EHR data to generate nutrition recommendations that were continuously adjusted as patient status changed. For example, TPN2.0 automatically increased sodium content when a newborn developed hyponatremia and then reverted after correction [83]. The TPN2.0 system used patient data to generate individualized TPN recommendations while maintaining physician oversight, illustrating how AI could support precision nutrition and therapeutic decision-making. This has the potential to improve nutrition care quality, especially in resource-limited settings.

### 6.4. Personalized Nutrition Interventions

There is a growing paradigm shift from generalized dietary recommendations towards personalized or precision-based nutrition strategies, driven by the ongoing rise in metabolic-related conditions like obesity, cardiovascular disease and type 2 diabetes [84,85]. Traditional dietary planning uses generalized population-level frameworks that overlook specific needs, whereas personalized nutrition encompasses individualized strategies that account for an individual’s genetic, metabolic, behavioral and environmental variability and work towards achieving health goals. ML has accelerated this transition towards personalized nutrition by enabling the integration and analysis of high-dimensional datasets and the development of predictive models capable of capturing non-linear relationships between diet and health outcomes.

While clinical medicine frequently makes generalizations based on population-based data (e.g., administration of a beta-blocker reduces blood pressure), understanding the relationships among clinical and biological variables provides insight into how a change in one influences the other. On the other hand, unlike population-based estimates, AI algorithms can map very high-dimensional relationships concurrently across an impressively large array of parameters to provide an intricate and detailed picture of individual-level profiles.

In silico representations of these data and/or generation of synthetic data can then serve as “digital twins” on which to test person-specific responses to interventions, such as diet [86]. Digital twin technology is an emerging application of AI that enables the creation of dynamic, virtual representations of individuals to simulate physiologic responses and optimize interventions. It has shown itself as a particularly promising application of AI in precision nutrition, as it integrates longitudinal clinical data, microbiome profiles, dietary intake and continuous monitoring to generate predictive simulations of disease progression and specific treatment outcomes.

Clinical application of digital twins has shown promise in metabolic disease outcomes. For example, in an open-label randomized trial, digital twins have shown promise compared to standard of care in reducing hemoglobin A1C levels and hyperglycemia in diabetic patients. Digital twin technology was used to predict individualized postprandial glycemic responses and suggest plans to optimize dietary strategies [87].

In critical care and nutrition settings, digital twins may be used to model energy expenditure, nutrient requirements, and metabolic stress responses, enabling highly individualized nutrition support strategies and implementing plans. Additionally, integration with CGM and wearable data allows for continuous model refinement, enhancing predictive accuracy over time. Digital twin technology may fill this gap in advancing personalized nutrition care for critical care patients. Notably, while digital twin technology presents incredible promise for boosting efficiency and precision in nutrition care and research, such technology is still in nascent stages of development.

Inflammatory bowel disease (IBD) is innately complex to manage, and AI-driven approaches via ML and DL have emerged as transformative in the management of IBD. AI is able to integrate and analyze multimodal diagnostic data, endoscopic images, magnetic resonance enterography, computed tomography and pathology slides to overall enhance diagnostic precision [88].

When it comes to precision or personalized nutrition in the context of IBD, there is significant room for advancement using AI. AI potentially addresses this limitation by using personalized data, guided by phenotypic, metabolic and microbiotic data, to develop individualized dietary plans to reduce intestinal inflammation and alleviate symptoms [89]. Genetic variations in individuals can influence someone’s ability to absorb nutrients and metabolism, resulting in often drastic dietary response differences.

AI has also been used in dietary assessments and nutrition tracking by estimating caloric and nutritional intake based on user interaction with digital platforms and even through computer vision of foods such as the goFOOD2 system [90]. Additionally, DL algorithms have demonstrated accuracy in detecting mucosal healing on endoscopic imaging in patients with ulcerative colitis; however, substantial heterogeneity was noted that may point to unreliability in diagnostic accuracy [91].

This structured mapping of individual data also supports the concept of precision nutrition. In the domain of gastroenterology, the use of AI for precision nutrition has been initially studied for irritable bowel syndrome (IBS). One example is the Heali app that scans food menu options or barcodes and leverages AI to provide personalized recommendations based on predefined dietary preferences.

A pilot RCT tested this app in 58 individuals with IBS, assigning them to either use the mobile app or receive educational materials over 4 weeks [92]. There was no statistical difference in reduction in the IBS symptom severity score between the two arms, although this was attributed to inadequate power from a modest sample size. The trial, nonetheless, demonstrated proof-of-concept functionality that could mimic, if not exceed, the provision of educational materials.

In a separate blinded pilot trial, 25 consecutive individuals with IBS were assigned to either an AI-assisted personalized diet or “standard IBS diet” for 6 weeks [93]. The AI-assisted diet involved an algorithm that identified micronutrients needed to modulate the gut microbiome toward more favorable profiles. The standard IBS diet included recommendations from the National Institute for Health and Care Excellence.

The investigators found significant improvement in IBS symptom severity scores in diet arms. Nonetheless, the degree of improvement of the overall score and its subcomponents was significantly greater for the AI-assisted diet when compared with the standard diet. The AI-assisted diet group also experienced a significant increase in beneficial microbes (e.g., Faecalibacterium and Propionibacterium), which were not seen in the control group.

Given these findings, the investigators conducted an RCT of 121 individuals with IBS, where participants were assigned to either receive the AI-assisted personalized diet or a low fermentable oligo-, di-, monosaccharide, and polyol (FODMAP) diet for 6 weeks [94]. Both diets were similarly effective in the reduction of IBS symptom severity scores, anxiety, depression, and quality of life measures. However, in subgroup analyses, the personalized diet had a significant reduction in IBS symptom severity scores for those with diarrhea-predominant IBS and significant improvements in IBS quality of life scores for those with diarrhea-predominant and mixed-type IBS; these were not significant for those on the low FODMAP diet.

Beyond using AI to design diet interventions, predictive modeling can help forecast therapy response. In a study of 308 children with Crohn’s disease who received exclusive enteral nutrition (EEN) for induction of remission, ML models were developed to predict early clinical remission and sustained corticosteroid-free remission after 36 weeks while on non-EEN maintenance therapy [95]. The models included 56 features related to diagnosis, EEN success or failure, follow-up visits, changes in anthropometrics and select laboratory values, and maintenance medications. AUROCs were modest, falling between 0.60 and 0.73 across models. In an unpublished study of an anti-inflammatory diet for induction of remission in Crohn’s disease, an extreme gradient boosting ML model was constructed to predict treatment response defined by a ≥50% reduction in fecal calprotectin from baseline and a value < 250 µg/g [96]. The AUROC was 0.62, roughly similar in performance to the predictive models for EEN response. While both models demonstrate proof of concept, further optimization would still be needed before they can be deployed in clinical settings.

AI prediction modeling can also be implemented to forecast outcomes. Using data among 879,730 malnourished patients hospitalized with IBD, decision tree-based ML models were able to predict mortality with good accuracy, sensitivity, and specificity of 0.99, 0.99, and 0.99 [97]. Such models could have benefit in flagging malnourished patients who could potentially benefit from more robust monitoring and intervention. Similarly, prediction models can be developed for other outcomes focused on gastroenterology and nutrition.

## 7. AI Applications for Clinical Support Tools and Documentation

### 7.1. Clinical Support Tools

AI-powered clinical tools such as OpenEvidence support clinical decision-making. OpenEvidence is an AI-powered platform that helps clinicians quickly access medical evidence [98]. It uses LLMs to search, synthesize, and summarize peer-reviewed research, guidelines, and medical literature. By translating large volumes of complex data into clear information, it functions as a real-time evidence assistant, improving efficiency and supporting informed clinical decisions. It is emerging as an alternative to traditional resources such as UpToDate [99], which have historically been used for vetted clinical information. It is noteworthy that UpToDate now has an “Expert AI” feature using an LLM that responds to queries based on their content, but the use of this feature currently requires an additional fee [100].

### 7.2. Clinical Documentation

The recent integration of AI in healthcare is rising, particularly in the domain related to clinical documentation [101]. It offers a promising solution to optimize clinical workflow and offset documentation burdens [102]. Accurate, efficient and comprehensive documentation is fundamental to high-quality patient care; however, documentation burden remains a large contributor to clinician fatigue and burnout, as administrative demands reduce time available for direct patient care [103]. With regard to clinical nutrition, documentation burdens are increased given the need for capturing nuanced nutrition histories, counseling and individualized care plans and are not well explored. AI uses are broad, encompassing ambient AI scribe systems (e.g., Nabla, DAX, Epic), LLMs, and EHR-integrated tools, which overall aim to increase efficiency and standardization within documentation but have their own limitations.

#### 7.2.1. AI Modalities in Clinical Documentation

AI in documentation has evolved from traditional speech recognition systems often involving considerable manual editing to advanced context-aware platforms capable of generating clinical narratives. Advances in natural language processing have enabled automated summarization and structured note generation, resulting in ambient AI systems and generative AI models that synthesize real-time conversations in the clinic setting and EHR data into coherent documentation [104].

Ambient AI scribe platforms such as Nuance DAX and Nabla utilize speech recognition and natural language processing to reduce manual documentation burden. One study noted that utilization of Nabla reduced time spent on notes compared to manual controls and overall improvement in burnout, task load and work exhaustion in both Nabla and DAX compared to the manual approach [104]. Similarly, Balloch et al. demonstrated that ambient AI tools improve documentation completeness and clarity while reducing clinician workload [105]. A large quality improvement multicenter study showed a reduction in burnout from 51.9& to 38.8% (odds ratio, 0.26; 95% CI, 0.13–0.54) after Abridge ambient AI implementation, with overall reduction in cognitive task load as well as increased perception that it could improve patient access to care and increase attention on patient concerns in an ambulatory environment [106]. Similarly, randomized and observational studies report reductions in documentation time of approximately 10–30%, with increased overall workflow efficiency [107].

#### 7.2.2. AI Clinical Documentation vs. Human-Generated Reports

LLMs such as Mistral-7B have been shown to generate concise and coherent discharge notes with comparable quality to physician-authored documentation [108]. Discharge summaries, in particular, represent a high-impact application due to their importance in care transitions as well as documentation burden and inherent time sensitivity. Further studies have shown that AI-generated discharge summaries were rated acceptable in 100% of cases compared with 92% of junior doctor summaries, and clinicians poorly distinguished AI-generated summaries from human-written notes (60% accuracy) [109]. Beyond discharge summaries, AI has shown utility in generating operative reports and documenting procedures. For example, neurosurgical applications show that AI-generated (ChatGPT) operative notes are highly coherent and efficient, though occasional inaccuracies do remain [110].

At the same time, it is understood that human-generated documentation simply remains the gold standard due to incorporation of clinical reasoning, lack of fabrication, contextual nuance and individualized patient concerns that are invaluable. Potentially, hybrid workflows would represent an optimal approach in terms of contextual nuance that clinicians offer and the efficiency that AI platforms model. Although this technology has already been deployed in clinical settings, larger and more robust research is still needed to confirm accuracy, efficiency, and practicality [104].

#### 7.2.3. Medicolegal and Ethical Considerations

The clinical adoption of AI-assisted documentation raises several medicolegal considerations that is critical to address alongside efficiency improvement prior to widespread implementation. First, patient confidentiality is a central concern, as ambient AI scribes and LLM-based tools process identifiable patient information sometimes via third-party servers, raising questions regarding the security of data obtained and compliance with HIPAA.

Second, AI-generated documentation requires rigorous verification prior to incorporation into the EHR. Clinicians may be inclined to accept AI-generated text without adequate review due to external factors such as time pressure. As such, there is a risk of undetected inaccuracies or fabricated content (i.e., hallucinations) being incorporated, thus giving potential for these errors to influence downstream clinical decision-making.

#### 7.2.4. Future AI Applications in Nutrition Documentation

As the aforementioned AI applications apply to nutrition-related documentation, these tools could inherently work to streamline nutrition-related history, examination and counseling documentation. Ambient AI scribe platforms have the ability to automatically extract important nutrition-related information such as caloric intake and dietary habits and can aid the documentation workflow without pauses in real-life clinical interactions. A potential role of AI also includes standardizing the documentation approach and modeling the NCP framework such that each component: assessment, diagnosis, intervention, and monitoring/evaluation becomes a mandated automatic prompt. This would thereby automate the documentation process and add consistency to documentation internationally.

## 8. Future Perspectives

The examples of AI in clinical nutrition care discussed in this review are summarized in Table 2. This table organizes the clinical applications based on their current implementation level and includes information about each application’s AI methodology, evidence type, validation status, and limitations. Overall, it shows that while a few technologies are approaching routine clinical use, many remain exploratory.

Future research in the area of AI in clinical care should move beyond proof-of-concept studies or model development and focus on clinically meaningful integration of AI tools in nutrition care. Several priorities stand out.

A first priority is establishing generalizability and comparability across studies. Prospective multicenter studies are needed to confirm that AI models can perform reliably across diverse patient populations and clinical settings. Development of standardized datasets and reporting methods would improve comparability between studies.

A second priority is demonstrating real-world value. Cost-effective analyses are needed to evaluate whether AI-assisted approaches justify their implementation relative to clinical benefit. Implementation science research should examine how AI tools can be feasibly integrated into improving clinical workflow efficiency, as well as barriers to integration and decision-making within real-world clinical practice. RCTs evaluating patient-centered outcomes, such as symptom control, quality of life, and long-term health, remain essential to establish genuine clinical benefit beyond technical performance metrics. Standardized human evaluation frameworks specifically developed for LLMs in healthcare that assess information quality, safety, and trustworthiness may further help ensure consistent assessment of generative AI tools as they apply to nutrition and dietary counseling [111].

A third priority concerns safe and responsible integration. Further work is needed to define how AI can be integrated safely into the NCP through human-in-the-loop models that preserve clinical judgment and oversight. Attention to transparency, bias, data security, and governance will also be essential, particularly as LLMs and other generative systems become more widely used in dietary counseling, documentation, and decision support.

**Figure 1:**
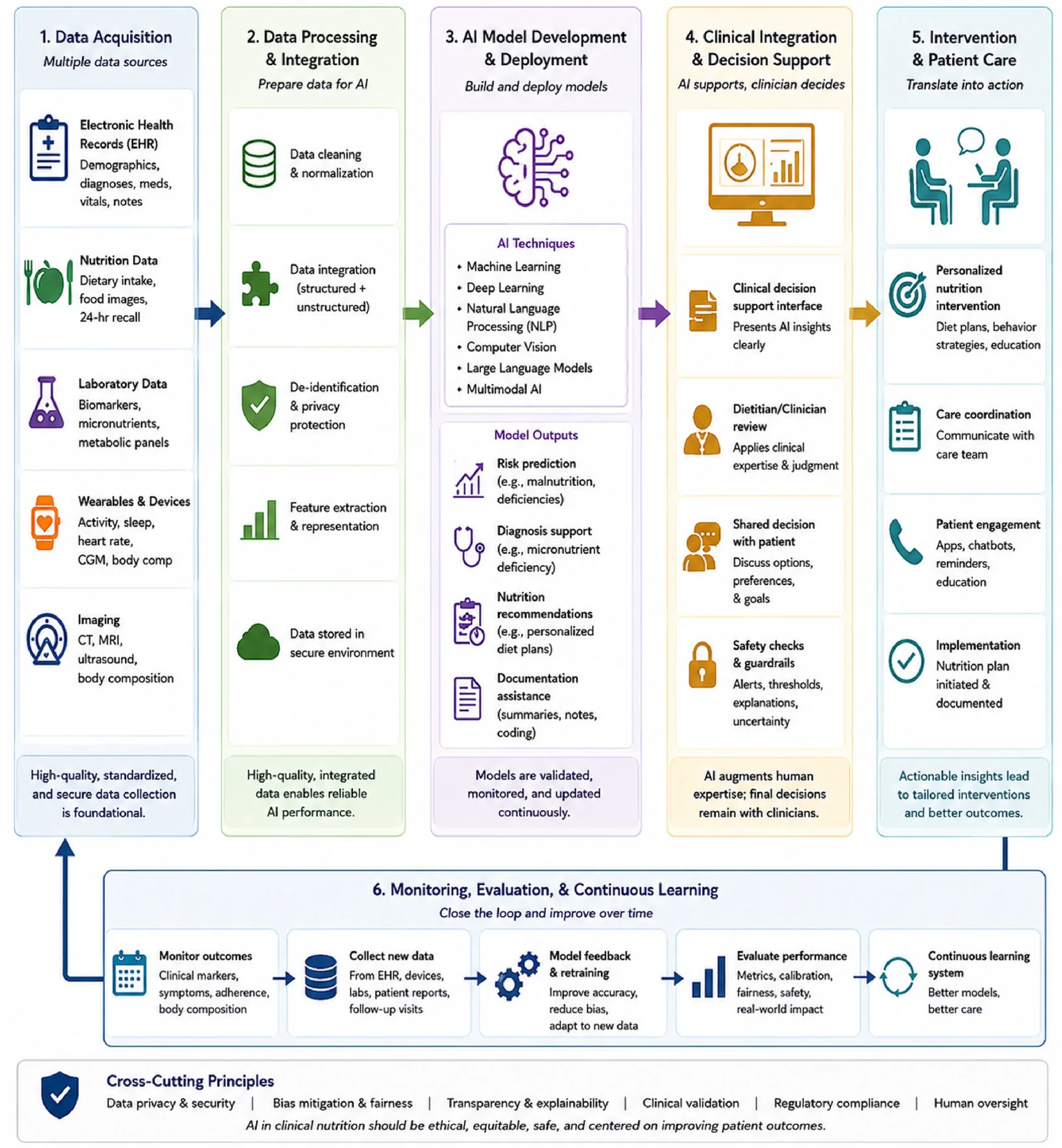
Conceptual workflow for AI integration into clinical nutrition care. Abbreviations and acronyms are listed at the end of this article.

Ultimately, the future of AI in clinical nutrition will depend not only on technical innovation but also on rigorous validation, thoughtful implementation, and alignment with the needs of patients and clinicians. Figure 1 represents a conceptual workflow illustrating how clinical data can be processed by AI systems to support nutrition assessment, diagnosis, intervention, monitoring and evaluation, and clinical decision-making. It outlines a theoretical implementation pathway and emphasizes that AI augments, rather than replaces, clinician judgment through continuous monitoring, validation, and human oversight.

## 9. Conclusions

As AI has permeated virtually all sectors and aspects of life, it holds substantial promise for nutrition. This narrative review discusses current and emerging applications of AI in clinical nutrition while focusing on the Nutrition Care Process. Within this framework, AI has exhibited benefits for nutrition assessment, monitoring, and evaluation, including semi-automated estimation of food intake, continuous assessment of biological and behavioral signals, and dynamic evaluation of nutrition-related disease risk. AI also offers potential for malnutrition screening and diagnosis, as well as non-invasive detection of nutrient deficiencies or excess.

Beyond assessment, AI may enhance nutrition-related interventions, such as personalized diet therapy, nutrition support, and eating behavior modification. AI’s capacity to integrate and analyze very large and complex datasets provides the ability to estimate response to therapy based on each individual’s unique characteristics, thus enabling optimized, tailored interventions. This feature also enables the development of AI-driven “digital twins” that could allow rapid simulation of effects of both existing and novel interventions in individuals with predefined characteristics.

Finally, AI can support clinical practice through natural language-based synthesis of the scientific literature, clinical documentation, and communication with patients.

Despite the expansive breadth of potential applications of AI in nutrition and healthcare, its use must be balanced with appropriate human oversight. AI should be viewed more as tools to augment, rather than replace, human-driven clinical care. Ultimately, responsibility and liability stemming from AI-assisted clinical decisions rest on the clinician, not the AI system.

Overreliance on AI can contribute to erosion of clinical skills, introduction of errors and bias, and potential harm to patients. Additionally, it is imperative to note that much of the evidence detailed in this review is derived from pilot studies, unpublished data not yet having undergone rigorous peer review, or single-center cohorts. Larger, prospective and externally validated studies, as prioritized above in the future perspectives section, are needed before firm conclusions can be made regarding the clinical impact of these tools.

In a study of 1443 patients undergoing colonoscopies without AI assistance, Polish investigators found that prior exposure to AI-assisted colonoscopies reduced the endoscopists’ ability to detect adenomas (−6.0%; *p* = 0.0089) after AI assistance was discontinued [112]. This “cognitive surrender” to AI can lead to reduced vigilance and ability to detect errors. Research has also demonstrated that automation can make anomaly detection and corrective intervention more difficult by undermining situational awareness, increasing system complexity, and obscuring the sources of error [113].

This latter phenomenon reflects a common criticism of AI: the black box nature of how it functions. That is, the pathways or methods for generating outputs are largely unknown to the user, thus limiting our ability to glean deeper insight into the relationships between individual variables and outputs, as well as the ability to understand what went wrong. Explainable AI methods that aim to make model outputs more clinician interpretable are a limitation that is an active area of research, as current approaches remain not fully tailored to clinical workflows [114,115].

In addition, AI model performance is constrained by the data used to train them. Gaps in source data may limit generalizability, while introducing unforeseen biases and errors. Retraining with higher quality data over time may improve performance. LLMs are also prone to hallucinations that lead to confident statements even when evidence is lacking or untrue. A further practical risk is susceptibility to hacking that could lead to unintended results [116]. Regulatory bodies have recently begun to address these concerns; for example, the U.S Food and Drug Administration has issued guidance on lifecycle management and predetermined change control plans for AI-enabled device software, reflecting this growing recognition of structured oversight needs [117,118].

In addition, given the significant computing resources required for some AI systems, data are often processed off-site; this opens risks to patient privacy during data transmission and processing. Moreover, as companies that build LLMs are strongly incentivized to harness real-world data to improve their models, clear agreements on how patient data are used are critical. There is ongoing debate on who owns the data, and regulations on the need for informed consent from patients when AI is used widely vary across regions. Nonetheless, there have been state- and national-level attempts to place guardrails on data and AI use, such as the California AI in Healthcare Act, European Union AI Act, and the United States Food and Drug Administration’s guidance on “software as [or in] a medical device” [118].

As AI capabilities continue to advance rapidly, potential and yet unimagined applications in nutrition will continue to blossom. In parallel, a recurring question is whether AI will ultimately replace human clinicians. While many people will increasingly rely on AI-assisted tools to answer their nutrition-related questions and manage routine needs, the role of human clinicians will likely remain for the foreseeable future, although evolving in how it functions. AI would serve as a powerful tool to augment and extend clinical care provided by humans.

Moreover, one of the “ironies of automation,” coined by the psychologist Lisanne Bainbridge, notes that automated systems can also increase human cognitive needs in other areas, such as the need for vigilance to ensure that critical problems do not develop or remain unaddressed. Finally, as much as thinking and communication can be simulated by AI, the empathetic human touch or interaction can never truly be replaced by the artificial machine.

## Figures and Tables

**Table 1:** General examples of AI use in healthcare.

Use Area	Examples
Clinical applications: diagnosis and risk prediction	Malnutrition screening [5,6,7,8], early kidney disease detection [9,10]
Clinical applications: treatment planning	Individualized dietary guidance based on -omics data [11,12]
Continuous health monitoring	Track biomarkers (blood glucose), physical activity, and other health markers such as dietary assessment over time [13,14,15]
Patient engagement	Supporting self-management through chatbots, virtual assistants, and personalized health education [16,17]
Clinical support uses	Documentation assistance, clinical decision-making support tools [18,19]
Research	Accelerate discovery and generate new clinical insights using pattern detection in large datasets [20,21]

**Table 2:** Summary analysis of examples of AI use in clinical nutrition care *.

Clinical Application	AI Methodology	Evidence Type	Validation Status	Limitations	Current Implementation
Clinical documentation	Ambient AI scribes (speech recognition, NLP); LLM-generated discharge summaries and operative notes [101,102,104,105,108,109,110]	RCT [104]; multicenter QI study [106]; multiple RCTs/observational studies [107]; comparative summary studies [108,109]	Documentation time reduced ~10–30% [107]; burnout reduced 51.9% to 38.8% (OR 0.26, 95% CI 0.13–0.54) [106]; AI discharge summaries rated acceptable in 100% of cases vs. 92% for junior doctors, with clinicians distinguishing AI from human notes only 60% of the time [109]	Occasional inaccuracies in generated notes [110]; human documentation remains the stated gold standard for clinical reasoning and nuance	Deployed across multiple health systems (Nabla, DAX, Abridge, Epic); nutrition-specific documentation in the framework of NCP still a future application
Clinical decision support AI tools	LLM-based medical literature synthesis platforms (OpenEvidence, UpToDate Expert AI) [98,99,100]	Platform description; no formal accuracy studies cited	None reported in this review	Assumption that information presented by platforms has been vetted; UpToDate’s AI feature is a paid add-on [100]	Already in clinical use as an alternative/adjunct to traditional resources like UpToDate [98,99]
Malnutrition screening	LLM feature extraction + gradient-boosted trees on EHR data [35]; ML-based MUST-Plus [38,39,40]	Retrospective cohort, *n* = 106,449 [35]; multicenter retrospective cohort [39,40]	AI outperformed nurse-administered MST (sensitivity 0.49–0.52 vs. 0.24–0.35, depending on outcome definition) [35]	Performance varies by outcome definition (discharge code vs. RD record) [35]	MUST-Plus already used in some hospital workflows, >90% usability among RDs, reduced diagnosis lag time [39]
Nutrition support, ICU/EN-TPN	ML prediction of feeding intolerance/diarrhea/refeeding hypophosphatemia [6,23,80]; ML for EN initiation [82]; TPN2.0 for neonatal TPN [83]	Model trained on >53,000 ICU patients [82]; TPN2.0 trained on 79,790 prescriptions/5913 neonates, externally validated on 63,273 prescriptions/3417 infants at a second institution [83]	Sepsis was the strongest predictor of EN initiation need [82]; TPN2.0 made appropriate automated adjustments (e.g., sodium correction for hyponatremia) on external validation [83]	Deployment outside study institutions not reported; study on neonates [83] not generalizable to other populations	TPN2.0 externally validated across two academic centers with physician oversight maintained, not autonomous [83]; other models remain research stage
Kidney disease (CKD, dialysis, AKI) management	Cardiac-data-based potassium detection without blood testing [11,30]; LLM-generated kidney-friendly recipes, menu translation, and food-image nutrient analysis [11]; AI-based alert systems for AKI/clinical deterioration risk [30,73,74]	Narrative descriptions; direct accuracy comparison reported only for the LLM dietary tool [11]	ChatGPT-4 underestimated calories by 36%, protein by 28%, potassium by 49%, and phosphorus by 54% compared to USDA-validated software [11]; no accuracy/validation figures for the potassium-detection or AKI alert systems themselves [30,73,74]	Nutrient-estimation errors from the LLM dietary tool could have serious clinical implications for renal patients [11]	Early stage; AI-generated dietary interventions explicitly stated to require RD review before implementation [11]; alert systems not described as deployed
Personalized/precision nutrition	Digital twins [87]; AI-personalized IBD/IBS diets [92,93,94]; treatment response prediction [95,96]; mortality prediction [97]; mucosal healing detection [91]	Open-label RCT [87]; pilot RCT *n* = 58 [92]; pilot *n* = 25 [93] RCT *n* = 121 [94]; cohort *n* = 308 [95]; cohort *n* = 879,730 [97]; meta-analysis [91]	Digital twin improved hyperglycemia vs. standard care [87]; AI-microbiome diet improved IBS symptoms in pilot [93] and performed similarly to low-FODMAP overall with subgroup benefit in diarrhea-predominant IBS [94]; Heali pilot found no significant difference, attributed to underpowering [92]; Crohn’s response AUROC 0.60–0.73 [95]; IBD mortality model sensitivity/specificity/PPV 0.99/0.99/0.99 [97]; mucosal healing DL showed substantial heterogeneity [91]	Underpowered pilot [92]; modest AUROC for response prediction [95,96]; heterogeneity limits mucosal-healing reliability [91]	Mixed; digital twin and AI-microbiome diet have RCT-level evidence, treatment-response and mucosal-healing models remain proof of concept
Eating disorders screening and treatment	Self-report tracking apps [61]; early warning system [62]; neuroimaging + ML [63,64]; reviews mentioning generative AI chatbot (Therabot) [76,77,78]	Validation study [62]; neuroanatomical study *n* = 30 [64]; Therabot RCT *n* = 210 [78]; clinician/community survey [79]	Early warning AUROC 0.92 vs. 0.89 in controls [62]; neuroimaging model 83.3% accuracy, *n* = 30 [64], support vector machine studies generally >80% accuracy [63]; Therabot RCT showed significantly greater symptom reduction at 4 weeks and 8-week follow-up [78]	Small sample sizes [64]; field needs further refinement [79]	Research/pilot stage; Therabot has the strongest evidence base but is not described as routinely deployed
Malnutrition diagnosis (imaging)	CT imaging to predict sarcopenia [42,43,44]; DL on ultrasound [45,46,47]	Small studies *n* = 100 [42], *n* = 51 [43]; Retrospective diagnostic accuracy study, *n* = 3096 [44]	Sensitivity 82.3%, specificity 98.1%, PPV 89.5% for CT model [44]; ultrasound shows only modest correlation with CT [47]	Ultrasound-CT correlation modest [47]; may not clearly outperform physical exam	Proof of concept; still requires on-site imaging
Micronutrient deficiency prediction	LLM symptom-based differential; ML/DL on routine labs (vitamin B12) [53]; DL on radiographs (rickets) [52]; Artificial neural network for iodine deficiency in pregnancy [54]; ML for vitamin D [55,56,57,58]	Cohort Study B12 *n* = 51,630 [53]; vitamin D *n* = 5106 [55] and *n* = 62,919 [56]; single studies for rickets [52] and iodine [54]	Vitamin B12: independent temporal validation cohort of 34,744, AUROC 0.88, sensitivity 0.92, specificity 0.67, PPV 0.73 [53]; rickets: sensitivity 80%, specificity 95% [52]; iodine: sensitivity 0.56, specificity 0.85 [54]; vitamin D models variable, one reporting near-perfect AUROC of 1.0 [56]	Iodine model requires data not routinely collected [54]; practical benefit limited by need for less accessible data	Proof of concept, mainly population-level risk stratification
Nutrient excess diagnosis	ML on demographic/lab data (hepatic steatosis) [59]; ML on sociodemographic/activity data (adolescent overnutrition) [60]	Single center, cross-sectional study [59]; retrospective study [60]	Hepatic steatosis: specificity of 94.6%, sensitivity of 45.2% (highest across models 68.0%) [59]; adolescent overnutrition AUROC 0.68–0.75 [60]	Modest sensitivity limits diagnostic use [59]; modest AUROC limits individual-level prediction [60]	Proof of concept
AI-assisted dietary/ nutrient intake assessment	Image recognition, wearable sensors, NLP [28]	Scoping review [28]; systematic review [29]	Food detection accuracy 74–99.85%, nutrient estimation error 10–15% [28]; AI estimates volume/calories about as well as, sometimes better than, humans [29]	Heterogeneous datasets and inconsistent reporting hinder comparison; authors call for standardized datasets [29]	Research and consumer app level, not a standardized clinical instrument
AI-driven analysis of continuous monitoring data	Predictive analytics applied to CGM/wearable data (arrhythmia, sleep apnea, depression, chronic disease detection [14]; prediabetes glucose pattern prediction [15]); ML cardiac-data-based potassium detection [30]	Scoping review [14]; narrative reviews [15,30]	Described as largely conceptual, with most accuracy data from small or single-center studies and no standardized databases or reporting methods [14]	Cost barrier, particularly in low-resource settings [14]; conceptual evidence base	AI analytics application is in early stage/emerging; underlying CGM/wearable hardware itself is already routine clinical technology

* Abbreviations and acronyms in table are listed at the end of the manuscript.

## Data Availability

No new data were created or analyzed in this review. Data supporting the findings of this review are available within the cited literature.

## Abbreviations

The following abbreviations are used in this manuscript:

AI	Artificial Intelligence
AKI	Acute Kidney Injury
AUROC	Area Under the Receiver Operating Characteristic Curve
BMI	Body Mass Index
CGM	Continuous Glucose Monitor
CT	Computed Tomography
DL	Deep Learning
EEN	Exclusive Enteral Nutrition
EHR	Electronic Health Record
EN	Enteral Nutrition
FODMAP	Fermentable Oligo-, Di-, Monosaccharide, and Polyol
GLIM	Global Leadership Initiative on Malnutrition
IBD	Inflammatory Bowel Disease
IBS	Irritable Bowel Syndrome
ICU	Intensive Care Unit
LLM	Large Language Model
ML	Machine Learning
MST	Malnutrition Screening Tool
MUST	Malnutrition Universal Screening Tool
NCP	Nutrition Care Process
NLP	Natural Language Processing
NRS-2002	Nutritional Risk Screening 2002
PPV	Positive Predictive Value
RCT	Randomized Controlled Trial
RD	Registered Dietitian
SGA	Subjective Global Assessment
TPN	Total Parenteral Nutrition
